# It Is Time to Shift Our Perspective to Focus on the Overall Health of Ostomy Patients Based on a Cross-sectional Study

**DOI:** 10.1097/DCR.0000000000003984

**Published:** 2025-12-08

**Authors:** Jiaqi Xu, Chen Pei, Qiaoling Sun, Xue Zhang, Ning Li, Qian Lu, Yujie Zhou

**Affiliations:** 1 Department of General Surgery, Peking University Third Hospital, Beijing, China; 2 International Medical Service, Peking University Third Hospital, Beijing, China; 3 International Medical Service, Peking University School of Nursing, Beijing, China

**Keywords:** Cross-sectional study, Ostomy care, Ostomy clinics, Wound ostomy and continence nurse

**See Editorial on page 313.**

## SURGICAL RESECTION WITH OSTOMY

Colorectal cancer (CRC) is one of the leading incident cancers. According to the GLOBOCAN 2022 estimates from the International Agency for Research on Cancer, the incidence and mortality of CRC ranked third and second among all cancers.^1^

Surgical resection with ostomy is a common treatment for these patients.^2,3^ It is estimated that approximately 100,000 ostomy surgeries are performed annually for patients with CRC in China, and the number is growing.^4^ Due to the change of defecation mode, survivors of cancer face a series of challenges, including physical, psychological, social, and spiritual problems.^5–7^ Among these challenges, the incidence of ostomy-related complications is not negligible and has been reported with a rate from 20% to 73.4%, which seriously affects patients’ quality of life.^8,9^

## WOUND, OSTOMY, AND CONTINENCE NURSES IN CHINA

Wound, ostomy, and continence nurses (WOCNs) are essential providers of ostomy care, providing health education and advanced treatment to improve patients’ quality of life and prognosis.^10^ Ostomy clinics led by WOCNs play a key role in providing continuous care, especially in managing stomal and peristomal complications, but the accessibility to these nurses may differ by location.^11^ For example, In China, although WOCN training was initiated in 2001,^12^ service coverage remains uneven across regions, with more than 70% of ostomy clinics concentrated in tertiary hospitals.^11^ Consequently, in areas with limited WOCN access, ostomy care is frequently delivered by nonspecialist clinicians (eg, general nurses, surgeons) with varying levels of specific training. This disparity in resource allocation presents significant challenges for ensuring consistent, high-quality ostomy care nationwide.

Within specialized ostomy clinics where WOCNs are present, WOCNs play a pivotal role. However, the current state of ostomy care practice by WOCNs in Chinese ostomy clinics remains unclear, including the scope of services and competence in managing stoma-related complications. Furthermore, although existing evidence-based guidelines and consensus statements emphasize holistic patient care,^4,13,14^ such statements offer insufficient specific guidance for complex clinical scenarios, and their implementation in Chinese WOCNs’ practice remains unclear.

Therefore, the purpose of this study was to evaluate the current status of WOCN nursing practice in ostomy clinics in China, identify existing problems or gaps, and optimize ostomy nursing services. An additional objective was to analyze the ability of WOCNs to manage stoma-related complications and to explore related factors, thereby providing a reference for supporting competency-based training programs.

### Sampling and Recruitment

This study was a cross-sectional survey. WOCNs in charge of ostomy clinics in China were recruited using purposive sampling in June 2024. Participants were included if they were registered nurses with WOCN training experience and were currently undertaking ostomy clinic work. The response size could not be calculated because there was no statistical information on the size of the target population in China and quantitative statistics on WOCNs’ competence in complication care.

### Questionnaire Development

According to relevant guidelines and group standards, we developed a questionnaire on nursing practice in the ostomy clinic. The questionnaire was reviewed and approved by 10 members of the Wound Ostomy Incontinence Nursing Committee of the Chinese Nursing Association (CNA). All members had more than 10 years of experience in ostomy care, held senior professional titles, and had a bachelor’s degree or higher. The scale-level content validity index was 0.979, and the item-level content validity index ranged from 0.8 to 1.0.

The questionnaire included the following:

- General nurse information, including age, professional title, years of experience in ostomy care, training experience, hospital level, and region.
- Ostomy care practices in ostomy clinics, including the frequency of outpatient services, annual outpatient volume, the work content, and the complications they were capable of managing.

With reference to the 11 stomal and peristomal complications outlined in "Nursing Care for Adult Enterostomy Patients,"^4^ a group standard by the Wound Ostomy Incontinence Nursing Committee of CNA, if all 11 complications can be managed in ostomy clinics, the WOCNs were considered to have high competence in complication care. Otherwise, the WOCNs were deemed to have low competence.

### Data Collection

The survey was conducted online, with electronic questionnaires distributed to hospitals across China through members of the Wound Ostomy Incontinence Nursing Committee of CNA. A total of 518 questionnaires were collected in this study. We excluded 191 invalid questionnaires based on the following criteria: 1) completion time <2 minutes, 2) obvious contradictions in answers, 3) not involved in ostomy outpatient service, and 4) not having WOCN training experience. Three hundred twenty-seven valid questionnaires were ultimately retained. The effective response rate was 63.1%.

### Data Analysis

SPSS 26.0 was used for the statistical description and analysis. Categorical data were expressed as frequencies (n) and percentages (%), whereas continuous data were presented as means and SDs. Univariate analysis of WOCNs’ competence in complication care was conducted using the χ^2^ test, and multivariate analysis was performed using binary logistic regression. A *p* value of <0.05 was considered statistically significant. There were no missing values for mandatory items in the questionnaire.

### Ethical Considerations

The study was conducted in accordance with the principles of the Declaration of Helsinki and was approved by the Ethics Committee of Peking University Third Hospital (approval No. IRB00006761-M2024451). Questionnaires were anonymous, and the submission of the questionnaire was regarded as consent to participate in the study.

### Participant Demographics

A total of 327 WOCNs from 269 hospitals in 28 provinces were investigated. Among them, 85.3% of the nurses received specialized training from enterostomal therapist schools or the Chinese Nursing Association, and 14.7% were trained by local provincial and municipal nursing associations. The mean age of the respondents was 42.0 ± 6.70 years (range, 28–60 years), and their mean experience in ostomy care was 10.5 ± 6.61 years (range, 1–40 years). Additional details are provided in Table 1.

**TABLE 1.** Demographics of WOCNs and univariate analysis of WOCNs’ competence in complication care(N = 327)

*Items*	*n (%*)	*Competence in complication care*		*χ^2^*	*p*
		*High competence group (N = 138*)	*Low competence group (N = 189*)		
Sex				0.403	0.525
Male	8 (2.4)	2	6		
Female	319 (97.6)	136	183		
Education level				1.989	0.370
Associate degree	7 (2.1)	4	3		
Bachelor degree	289 (88.4)	118	171		
Master degree	31 (9.5)	16	15		
Profession title				2.515	0.284
Junior	10 (3)	4	5		
Intermediate	170 (50.7)	63	103		
Senior	155 (37)	71	81		
Management position				0.826	0.364
No	169 (50.5)	58	89		
Yes	144 (43)	80	100		
Years of experience in ostomy care				14.999	0.001
<5 y	53 (16.2)	15	38		
5–10 y	106 (32.4)	35	71		
≥10 y	168 (51.4)	88	80		
Engaged full-time in ostomy care				3.399	0.065
No	222 (67.9)	86	136		
Yes	105 (32.1)	52	53		
Received continuing education for ostomy care in the past 5 y				7.951	0.005
No	47 (14.4)	11	36		
Yes	280 (85.6)	127	153		
Learned the standards or guidelines on ostomy care				7.881	0.005
No	41 (12.5)	9	32		
Yes	286 (87.5)	129	157		
Type of hospital				0.270	0.603
General	297 (90.8)	124	173		
Specialist	30 (9.2)	14	16		
Level of hospital				7.329	0.007
Tertiary	296 (90.5)	132	164		
Secondary-level	31 (9.5)	6	25		
Region of hospital				9.653	0.002
City-level	263 (80.4)	122	141		
County-level	64 (19.6)	16	48		
Regular certification or assessment for WOCNs				4.654	0.031
No	165 (50.5)	60	105		
Yes	162 (49.5)	78	84		
Ostomy outpatient management system used				12.523	<0.001
No	35 (10.7)	5	30		
Yes	292 (89.3)	133	159		
Regular case analysis of complex ostomy cases				26.557	<0.001
No	124 (37.9)	30	94		
Yes	203 (62.1)	108	95		

WOCN = wound, ostomy, and continence nurse.

## STATUS OF OSTOMY CARE PRACTICE IN OSTOMY CLINICS

### Overview of Outpatient Ostomy Care

Among 327 WOCNs, 252 (77.1%) were capable of independently conducting ostomy clinic visits. The most common frequency of ostomy clinic sessions was once per week (46.5%; 152/327). The work content of the ostomy clinics primarily included managing related complications and routine ostomy care (100%), followed by providing health education (97.6%; 320/327) and psychological counseling (92.4%; 302/327). During patient follow-up visits, WOCNs focused more on stomal and peristomal complications (99.1%; 324/327), patients’ or caregivers’ knowledge and skills in ostomy self-care (95.4%; 312/327), and risk factors for the complications (94.2%; 308/327). Relatively less attention was given to physical and social well-being concerns, with sexual and intimacy concerns being the least addressed (33.3%; 109/327). Additional details are provided in Table 2.

**TABLE 2.** Overview of outpatient ostomy care (N = 327)

*Items*	*n* (*%*)
Independently conducting enterostomy clinic services	
No	75 (22.9)
Yes	252 (77.1)
Frequency of ostomy clinic sessions per week	
<1	20 (6.3)
1	152 (46.5)
2-4	103 (31.5)
≥5	52 (15.9)
Outpatient service workload per year	
<300	160 (48.9)
300–1000	72 (22.0)
≥1000	95 (29.1)
Work content of the ostomy clinics^a^	
Treating or managing related complications	327 (100)
Routine enterostomy care	327 (100)
Health education	320 (97.6)
Psychological counseling	302 (92.4)
Provision of stoma supplies	288 (88.1)
Complications treated or managed in ostomy clinics^a^	
Peristomal skin injury	322 (98.5)
Mucocutaneous separation	301 (92.0)
Stomal edema	289 (88.4)
Stomal bleeding	282 (86.2)
Stomal retraction	272 (83.2)
Peristomal granuloma	255 (78.0)
Stomal stenosis	252 (77.1)
Stomal prolapse	244 (74.6)
Parastomal hernia	235 (71.9)
Peristomal folliculitis	215 (65.7)
Stomal ischemia/necrosis	213 (65.1)
Factors to consider in the selection of stoma supplies^a^	
Type and location of stoma	327 (100)
Body and stoma profiles	323 (98.8)
Lifestyle	289 (88.4)
Eyesight and hand dexterity	281 (85.9)
Patient preference	258 (78.9)
Patient issues focused on during follow-up visits^a^	
Stoma-related issues	
Stomal and peristomal complications	324 (99.1)
Patients’ or caregivers’ knowledge and skills in ostomy self-care	312 (95.4)
Risk factors for complications	308 (94.2)
Physical and social well-being concerns	
Diet and nutritional needs	298 (91.1)
Physical activity	270 (82.6)
Psychological health	251 (76.8)
Sleep problems	235 (71.9)
Social/interpersonal relationships and returning to working life	228 (69.7)
Travel	200 (61.2)
Social support	198 (60.6)
Intimacy and sexuality	109 (33.3)

WOCN = wound, ostomy, and continence nurse.

a Multiple responses questions. All other questions required a single choice.

### Competence of WOCNs in Managing Stomal and Peristomal Complications

In terms of competence in treating or managing complications, peristomal skin injury (98.5%; 322/327) and mucocutaneous separation (92.0%; 301/327) accounted for the highest proportions (see Table 2 for details). Among the WOCNs, 42.2% had high competence in complication care (ie, capable of handling or participating in the treatment of all 11 complications surveyed), whereas 57.8% showed low competence (ie, inability to manage at least 1 complication).

Univariate analysis of WOCNs’ competence in complication care revealed that several factors significantly influenced their competence. These included individual-level factors such as years of experience in ostomy care (*p* = 0.001) and whether one received continuing education in ostomy care in the past 5 years (*p =* 0.005) or learned the standards or guidelines on ostomy care (*p* = 0.005). Organizational-level factors included the level of hospital (*p* = 0.007), the region of hospital (*p* = 0.002), regular certification or assessment for WOCNs (*p* = 0.031), whether the WOCN had access to an ostomy outpatient management system (*p* < 0.001), and regular case analysis of complex ostomy cases (*p* < 0.001). Details are provided in Table 1.

Multivariate analysis was performed using binary logistic regression analysis. The assignment of independent variables is shown in Table 3. The regression analysis results indicated that 10 years or more experience in ostomy care (OR 2.312; 95% CI, 1.110–4.812; *p* = 0.025) and regular case analysis of complex ostomy cases (OR 2.014; 95% CI, 1.159–3.499; *p* = 0.013) were independent factors for WOCNs’ competence in managing complications (*p* < 0.05). Details are provided in Table 4.

**TABLE 3.** Variable assignments

*Items*	*Variable assignments*
Dependent variable	
WOCNs’ competence in complication care	0 = Low competence group 1 = High competence group
Independent variables	
Years of experience in ostomy care	1 = <5 y 2 = 5–10 y 3 = ≥10 y
Whether received continuing education for ostomy care in the past 5 y	0 = no, 1 = yes
Whether learned the standards or guidelines on ostomy care	0 = no, 1 = yes
The level of hospital	0 = secondary-level hospital 1 = tertiary hospital
The region of hospital	0 = county-level hospital 1 = city-level hospital
Regular certification or assessment for WOCNs	0 = no, 1 = yes
Ostomy outpatient management system used	0 = no, 1 = yes
Regular case analysis of complex ostomy cases	0 = no, 1 = yes

WOCN = wound, ostomy, and continence nurse.

**TABLE 4.** Factors related to WOCNs’ competence in complication care

*Independent variables*	*OR*	*95%* *CI*	*p*
≥10 y of experience in ostomy care (ref: <5 y)	2.312	(1.110–4.812)	0.025
Regular case analysis of complex ostomy cases (ref: no)	2.014	(1.159–3.499)	0.013
5–10 y of experience in ostomy care (ref: <5 y)	1.197	(0.555–2.580)	0.646
Received continuing education for ostomy care in the past 5 y (ref: no)	1.828	(0.847–3.946)	0.124
Learned the standards or guidelines on ostomy care (ref: no)	1.921	(0.838–4.406)	0.123
Ostomy outpatient management system used (ref: no)	2.646	(0.936–7.483)	0.067
Regular certification or assessment for WOCNs (ref: no)	1.409	(0.856–2.320)	0.178
Tertiary hospital (ref: secondary-level hospital)	1.225	(0.405–3.708)	0.719
City-level hospital (ref: county-level hospital)	1.836	(0.894–3.771)	0.098

WOCN = wound, ostomy, and continence nurse.

## CURRENT PRACTICES IN CHINA

Our study was the first to investigate current practices among WOCNs in ostomy clinics at secondary-level and tertiary-level hospitals in China. We collected comprehensive data on WOCNs’ outpatient workload, work contents, competence in complication care, and patient issues addressed during follow-up visits in ostomy clinics. We identified existing clinical practice issues and provide references to improve the quality of ostomy clinic care services.

This study shows that the work content of WOCNs in ostomy clinics in China is comprehensive, encompassing the management of stoma-related complications, routine care, health education, psychological counseling, and the provision of stoma supplies. Stomal leakage is one of the primary concerns for ostomy patients.^15^ Repeated leakage increases the risk of peristomal skin complications and exacerbates psychological burdens such as anxiety, fear, and impaired self-esteem, while also limiting patients’ social interactions and daily activities, significantly affecting their quality of life.^16^ Indrebø et al^17^ found that a significant association exists between having a good relationship with a health professional and a lower frequency of leakage. WOCNs can identify leakage early and analyze the causes, providing targeted education or treatment strategies to reduce the recurrence of leakage and the risk of related complications. In this study, we also found that Chinese WOCNs focus more on patients’ knowledge and skills in ostomy self-care, stoma-related complications, and their risk factors during follow-up visits. They can provide services such as managing stoma-related complications, providing routine care and health education, and addressing patients’ physiological needs related to disease diagnosis and treatment.

In this study, we also identified certain deficiencies in the ostomy care practice of WOCNs in ostomy clinics. First, WOCNs paid insufficient attention to the overall health issues of ostomy patients during follow-up visits, especially regarding sleep problems, social functioning and support, travel concerns, and sexual and intimacy concerns. With the increasing incidence of CRC among younger populations and the extended survival of ostomy patients, CRC survivors have more diverse postoperative rehabilitation needs.^18^ WOCNs should not only focus on the local health of the stoma but also provide guidance on patients’ diet, physical activity, psychological well-being, and stoma-related considerations (eg, intimacy, travel, social interactions). This aligns with the World Council of Enterostomal Therapists International Ostomy Guideline 2020, which underscores a holistic approach as essential.^13^ Zhang et al^19^ also demonstrated that holistic care can effectively improve patients’ stoma adaptation and alleviate psychological distress. Particularly for patients with a temporary diverting stoma, early interventions in nutrition, rehabilitation, and psychological support may positively impact functional recovery after ostomy closure.

Second, we found significant variability in the competence of Chinese WOCNs in managing stoma-related complications. Based on "Nursing Care for Adult Enterostomy Patients,"^4^ we evaluated WOCNs’ reported ability to manage 11 stoma-related complications in clinical settings, including peristomal skin injury, mucocutaneous separation, stomal edema, bleeding, retraction, and so on. The results showed that only 42.2% of WOCNs reported that they were capable of managing all 11 complications. The highest proportion (98.5%) was peristomal skin injury, whereas the lowest proportions (both below 70%) were peristomal folliculitis and stomal ischemia/necrosis. Although guidelines and expert consensus on stoma-related complications are available both domestically and internationally,^13,20^ the clinical differentiation and diagnosis of various complications, particularly peristomal complications, remain challenging. In the future, targeted training programs should be developed to enhance WOCNs’ competence in complication care. Furthermore, for the diagnosis and treatment of complex and severe complications, WOCNs need to strengthen multidisciplinary collaboration with dermatology, surgical oncology, clinical nutrition, and other specialties to promptly solve complex or potential comprehensive health problems in patients.^11^

Furthermore, we analyzed factors related to the competence of Chinese WOCNs in the care of patients with complications. The results indicated that having more than 10 years of experience in ostomy care and regular case analysis of complex ostomy cases were independent factors. Case analysis of complex cases is a form of case-based learning (CBL), a teaching method commonly used in medical education that is modeled on patient presentations and the subsequent workup of a condition and the course of presentation, diagnosis, treatment, and follow-up.^21^ The effectiveness of CBL has been demonstrated across various disciplines, encompassing learners from various educational levels and backgrounds.^22–24^ Using the strengths of CBL, it can integrate case information such as images of complex ostomy cases, patient treatment processes, decision-making, and outcomes with key knowledge. Although case analysis was associated with higher competence in our study, the effectiveness of CBL as an educational intervention for WOCNs warrants further empirical investigation.

### Strengths and Limitations

Regarding the strengths of this study, we had a wide range of respondents from 269 hospitals across 28 provinces in China. The self-designed questionnaire was reviewed by authoritative experts and demonstrated good content validity. However, there were some limitations in this study. First, convenience sampling was used because of practical constraints, potentially limiting generalizability. Second, self-reported measures have inherent risks of recall bias and/or social desirability bias. Third, although this study identified that work experience and case analysis were correlated with competence, the cross-sectional design precludes causal inference; nevertheless, these findings provide preliminary evidence supporting competency-based training programs among WOCNs and nonspecialist clinicians. Fourth, this study used simplified indicators to assess the competence of WOCNs in complication care; consequently, a more detailed investigation of their knowledge, attitudes, and practices regarding complication care is necessary to refine advanced training curricula.

### Recommendations for Further Research

1. The patient version of guidelines urgently needed for ostomy patients’ healthy living should cover self-management and critical health-related aspects, such as diet, physical activity, sleep, travel, intimate relationships, and social interactions.
2. Future research could integrate online-to-offline models using artificial intelligence technologies. These models would combine mobile health and patient versions of guidelines with ostomy clinics to deliver holistic nursing care for ostomy patients, thereby enhancing their quality of life.

### Implications for Policy and Practice

For settings with WOCN access, regional and hospital administrators should leverage the comprehensive health management capabilities of ostomy clinics to enhance multidisciplinary care for patients with CRC. Through ostomy clinics, systemic health issues in patients can be identified and prevented, and timely referrals to nutrition, rehabilitation, and mental health services can be made when needed. WOCNs should strengthen the concept and skills of holistic care in ostomy clinics.

In resource-limited settings, clinical practice guidelines and specialized training are vital resources, enabling nonspecialist clinicians to deliver essential ostomy care, manage basic complications, and identify referral needs—thus bridging service gaps. To further enhance ostomy care competencies, it is recommended that CBL be implemented in departmental training programs for nonspecialist clinicians. Particularly essential is multidisciplinary analysis of complex cases involving multimorbidity, challenging stoma-related complications, and other health conditions. Such targeted training fosters treatment consensus and elevates holistic care quality. In addition, we propose that, were it developed, a patient guideline for healthy living could be used as a tool to advance holistic care.

## CONCLUSIONS

The work content of Chinese WOCNs in ostomy clinic care is comprehensive, encompassing the management of stoma-related complications, routine care, health education, and other services. However, WOCNs should increase their focus on and guidance for the overall health of ostomy patients during follow-up visits. In addition, it is necessary to promote CBL-based training programs in departments to further enhance WOCNs’ competence in complication care.

## ACKNOWLEDGMENTS

The authors thank the experts who participated in the development of the questionnaire and all the respondents who participated in the survey.
